# Mr. Evans' Amalgam for Filling Teeth

**Published:** 1849-07

**Authors:** 


### Miscellaneous Notices.

*Mr. Evans' Amalgam for Filling Teeth.*—It has been frequently stated to us, that Mr. Evans, an American dentist, now associated in the practice of his profession with Dr. Brewster of Paris, had, since leaving this country, become an advocate of the use of amalgam for filling teeth. We also received, some time since, a number of the London Lancet, containing a communication from this gentleman, proposing a new combination of mercury, which he thought free from the objections that the various other amalgams, that had been employed for this purpose, evidently possessed. In a subsequent number of the above mentioned journal, Mr. Levison, an English dentist, replied to the communication of Mr. Evans, objecting, on what he conceived to be good grounds, to the use of his amalgam. The last article, as our readers will recollect, we republished in the journal, and as this gave the composition of the new amalgam, we did not deem it necessary to copy the first.

The communication above referred to, induced us to place some credence in the statements which had been made to us in reference to Mr. Evans' practice in Paris. Still we were unwilling to believe that a man possessed of his skill in the operation of filling teeth with gold, could be induced to use amalgam for any other purpose than as an experiment, and we are gratified to learn, as will also be his professional friends in the United States, that our opinion was correct.

As Mr. Evans has been so kind as to send us some of his new amalgam, with a request that we would try it and favor him with our opinion with regard to its value, it will give us pleasure to submit it to such tests as we may deem necessary to enable us to form a correct judgment in relation to its fitness for filling teeth. In the mean time, we would caution him against experimenting with it to an extent that would be likely to injure the reputation he has it in his power to form in the great French metropolis—the field which he has selected for his professional labor.

Mr. Evans is a young practitioner of high promise, and as he says he has never seen a tooth which could not be filled with gold that could be preserved—and believing, from the evidences of his skill we have seen, that he can fill any such tooth with gold—we should regret to have him throw away the reputation he has acquired for excellence in this operation, by tampering too extensively with an article—to say the most of it—of doubtful value. In the hands of skilful practitioners, we believe no better material for filling teeth than gold can be had; and if the object of this operation can be fully accomplished with this metal, why seek a substitute? If a tooth is worth filling at all, it is worth filling with the best material, and in the very best manner. If it can be ascertained that Mr. Evans' new amalgam is as good as gold, we shall not object to its use. On the contrary, we shall hail it as one of the most important discoveries that has ever been made in dental surgery, and the author of it as one of the greatest benefactors to mankind. We sincerely hope it may prove to be so, as the operation of filling teeth, the proper performance of which at present requires so much labor and nice manipulation, would then be rendered very simple and easy.

We ask for Mr. Evans' letter an attentive and careful perusal. We do not know that it was designed for publication; but we think it due to him and his friends in this country, that his views upon the subject should be fully known.—*Balt. Ed.*

PARIS, *May* 10*th*, 1849.

MY DEAR SIR:—I feel desirous of its being known, that in giving this amalgam to the profession to test, I do not advocate the use of amalgams. I *never* have, in *any* case, used *any* of the compounds hitherto so much in use. I have always said, and still repeat, that I never have seen the tooth which I could not fill with *gold*, if it could be preserved at all.

I have made many experiments in metals and other substances, with a view of discovering something to supplant the many bad oxydizable substances which are so generally used in America, and much more extensively in Europe. There are many in London, who are esteemed by me, and of whose acquaintance I am proud, that use, in some cases, these substances, for the want of better—believing they do the best for their patients under the circumstances. Unfortunately, there are too many who use them indiscriminately, not possessing sufficient skill or judgment.

I hope this article may be well tested, and if found in the slightest injurious, I shall feel it a favor upon the part of my professional friends, to condemn it. The moment I am convinced of its producing any injurious effect, I shall, in the strongest terms, deprecate its use—for I consider the man who will make use of anything in his profession, that has a deleterious effect upon the teeth or general health, and he *knowing* it, is not worthy to be regarded as a member of our important branch of the medical profession.

The conviction that this substance is not productive of harm, from the fact that it does not oxydize, but retains its color perfectly, has caused me to countenance any experiments of the kind—as yet I have only used it experimentally. Also, from the fact, that a gold band or clasp, being placed in contact with it, does not become touched by the mercury—consequently not destroyed, as is the case with the ordinary amalgams.

You will pardon the liberty I have taken in thus addressing you, but presuming you have received the "London Lancet," 21st April, which contains my communication, I felt that it demanded a slight explanation from me, as I do not wish to be classed among those who advocate the use of amalgams as hitherto composed.

A letter from you, when time permits, will be gladly received by

Yours, respectfully,

THOS. W. EVANS.

15 RUE DE LA PAIX, PARIS.

